# Antenatal care utilization on low birth weight children among women with high-risk births

**DOI:** 10.12688/f1000research.126814.1

**Published:** 2023-04-13

**Authors:** Diah Puspita Sari, Mario Ekoriano, Resti Pujihasvuty, Sari Kistiana, Sri Lilestina Nasution, Irma Ardiana, Edy Purwoko, Yuli Puspita Devi, Muthmainnah Muthmainnah

**Affiliations:** 1 National Research and Innovation Agency [BRIN], Jakarta, Indonesia; 2 National Population and Family Planning Board [BKKBN], Jakarta, Indonesia; 3 Faculty of Public Health, Universitas Indonesia, Depok, Indonesia; 4 Faculty of Public Health, Universitas Airlangga, Surabaya, Indonesia

**Keywords:** antenatal care, risky maternal, low birth weight, pregnant women

## Abstract

**Background:** Low birth weight (LBW) is a major public health problem in Indonesia, while LBW is a leading cause of neonatal mortality. Adequate antenatal care (ANC) utilization would help to prevent the incidence of LBW babies. This study aims to examine the association between ANC utilization and LBW children among women with high-risk birth criteria. High-risk birth criteria consisted of 4T which were too young (mother’s age <20 years old), too old (mother’s age >35 years old), too close (age gap between children <2 years), and too many (number of children >2 children).

**Methods:** This study utilized calendar data from the women’s module from the 2017 Indonesia Demographic and Health Survey (IDHS), with the unit analysis only the last birth of women of childbearing age (15–49), which numbered 16,627 women. From this number, analysis was done by separating the criteria for women with high-risk birth. Multivariate logistic regression analyses were employed to assess the impact of ANC and socio-demographic factors on LBW among women with high-risk birth criteria.

**Results:** This study revealed that only among women with too many children criteria (>2 children), adequate ANC utilization was significantly associated with LBW of children, even after controlling for a range of socio-demographic factors (p < 0.05). In all four women criteria, preterm birth was more likely to have LBW than those infants who were born normally (above and equal to 2500 grams) (p < 0.001).

**Conclusions:** According to WHO, qualified ANC standards have not been fully implemented, including in the case of ANC visits of at least eight times, and it is hoped that ANC with health workers at health facilities can be increased. There is also a need for increased monitoring of pregnant women with a high risk of 4T to keep doing ANC visits to reduce LBW births.

## Introduction

One of the focuses of the National Mid-Term Development Plan (RPJMN) in 2020–2024 was reducing the maternal mortality rate (MMR) and infant mortality rate (IMR). ^1^ Neonatal mortality rate (NMR) and IMR are indicators of child mortality, this figure shows an improvement since 1990. NMR decreased from 20 per 1,000 live births in 2002 to 15 per 1,000 live births in 2017 and IMR from 35 per 1,000 live births in 2002 to 24 per 1,000 live births in 2017. ^2^ However, this figure still has not reached the 2024 target, where NMR is expected to decrease to 10 per 1,000 live births and IMR to 16 per 1,000 live births. ^1^

The main causes of neonatal death in developing countries include low birth weight (LBW) and premature birth. Data showed that LBW and premature births were 19% in 2016. ^3^ Babies with LBW are defined by WHO as babies born less than 2,500 grams regardless of gestational age. ^4^ In Indonesia, the percentage of LBW has decreased slowly, from 11.2% in 2000 to 10.2% in 2012 then to 10.0% in 2015. ^4^

Babies with LBW have a higher risk of stunting, low intelligence (IQ), and death in the first 28 days of life. ^4^^,^^5^ In addition, the risk of death at the age of under 1 year is 17 times greater than that of infants with normal birth weight. ^6^ In adulthood, infants with LBW are at risk for obesity, heart disease, and diabetes. ^4^

LBW can be caused by premature birth (<37 weeks), babies with small gestational age (SGA), or a combination of both. ^4^^,^^7^ The lower the gestational age, the lower the baby’s birth weight automatically because physiologically and anatomically the fetal organs have not grown and developed perfectly, and the risk of illness and death will increase. ^8^

Premature births and fetuses that fail to thrive in the womb are influenced by four maternal factors, namely maternal malnutrition, maternal health problems during pregnancy, maternal characteristics, and other factors. ^4^ In addition, obstetric factors such as maternal age, both too young and too old, significantly affect LBW. ^9^^,^^10^

Pregnancy in women with “4T *,*” namely “too young (gave birth <20 years), too old (gave birth >35 years), too close (short birth gap), and too many (a large number of children)” can have a positive effect on both the mother and the fetus being born. ^8^^,^^11^^–^^15^ This risk can be prevented or minimized by performing qualified antenatal care (ANC). ANC is important to prevent, detect and treat maternal and fetal health problems. ^3^^,^^8^^,^^11^^,^^12^^,^^16^^–^^18^

Since 2016, WHO has recommended pregnant women to have a minimum of eight pregnancy check-ups. ^19^ WHO provides guidance for pregnant women to have a healthy pregnancy (positive pregnancy) through five interventions and 19 recommendations as well as several recommendations for specific cases.

Since 2020, it is agreed in Indonesia for pregnant women to make ANC visits at least six times, with at least two contacts with doctors in the first trimester to screen for risk factors/pregnancy complications; and in the third trimester for one-time delivery risk factor screening. Based on IDHS in 2017, National Family Planning stated that the coverage number of ANC visits (>4 times) in Indonesia was 90.6% and as many as 75% carried out pregnancy checks by health workers. ^2^

The difference between this study and other similar studies is to look at the effect of ANC on women with 4T with the incidence of LBW. Therefore, this study aimed to determine the effect of ANC on women with 4T on the incidence of LBW. The hypothesis that is built is that qualified ANC in women of childbearing age with 4T reduces the risk of LBW events.

## Methods

### Study design

This study used the 2017 Indonesian Demographic and Health Survey (IDHS) calendar data source, the women of childbearing age module. This study is mostly retrospective data, which requires each respondent to report their experience in ANC at the time of pregnancy and birth history. This study analyzed 49,627 women of childbearing age (15–49 years) with a total of 16,627 last births because the LBW number available in the IDHS was the last birth history.

The criteria for the unit of analysis were “4T”, among others; “too young” (gave birth <20 years) totaled 843; “too old” (gave birth >35 years) totaled 2,530; “too close” (spacing of fewer than two years) totaled 5,300; and “too many” (bearing more than two children) totaled 873.

The independent variables being analyzed were ANC quality, area of residence, education level, wealth level, work status, ANC examination place, ANC examiner staff, and access to information media. The qualified ANC indicator in the WHO guidelines is positive pregnancy. ^19^ There were only five recommendations that allow for analysis, namely getting iron, getting bacteria in the urine, getting tetanus toxoid (TT) injections during pregnancy, visiting at least eight ANC, and screening of smoking history. The dependent variable was the incidence of LBW in women with 4T.

### Data analysis

The data analysis of this study used the IBM SPSS application version 21. The analysis of this study was carried out descriptively and inferentially. Descriptive analysis through univariate and bivariate analysis was conducted to determine the frequency distribution of the variables studied. Inferential analysis was carried out through multivariate analysis with binary logistic regression models (crude OR and adjusted OR) to determine the effect of the independent variables on the dependent variable.

### Ethics statement

According to the DHS Program, “the procedures and questionnaires for standard DHS surveys are reviewed and approved by The Institutional Review Board (IRB) of ICF International while country-specific DHS protocols are reviewed by the IRB of ICF International and typically by an IRB in the host country”. The IRB of ICF International ensures the protection of human subjects from the survey complies with the U.S. Department of Health and Human Services regulations, while the host country IRB ensures that the survey complies with the laws and norms of the nation. While downloading the data, the names and addresses of the respondents are de-identified. The data have been obtained by registering and requesting with the Demographic and Health Surveys (DHS) website ( https://dhsprogram.com).

## Results

The results of the univariate analysis presented a description of social, economic, and demographic characteristics as shown in Table 1. Descriptively, women in this study were relatively more middle-educated in each category (69% “too young”, 52% “too close”, 49% “too many”, and 45% “too old”). Based on the area of residence, the majority of women living in rural areas were “too young” (66%), “too many” (53%), and “too close” (51%), while women with “too old” (> 35 years) live more in urban areas (51%) than in rural areas (49%).

**Table 1.** Sociodemographic characteristics of women with 4T.

Variables	Too young (N=843) n (%)	Too old (N=2530) n (%)	Too many (N=5300) n (%)	Too close (N=873) n (%)
**Education level**				
Low	252 (29.9)	1.092 (43.2)	2.181 (41.1)	238 (27.3)
Middle	579 (68.7)	1.126 (44.5)	2.574 (48.6)	457 (52.3)
High	11 (1.3)	313 (12.4)	545 (10.3)	178 (20.4)
**Region**				
Urban	288 (34.2)	1.297 (51.2)	2.517 (47.5)	430 (49.2)
Rural	555 (65.8)	1.234 (48.8)	2.784 (52.5)	443 (50.8)
**Wealth index**				
Low	502 (59.6)	991 (39.2)	2.345 (44.2)	410 (46.9)
Middle	191 (22.6)	476 (18.8)	990 (18.7)	141 (16.2)
High	150 (17.8)	1.063 (42.0)	1.965 (37.1)	322 (36.9)
**Employment status**				
Not working/housewife	581 (68.9)	1.216 (48.1)	2.627 (49.6)	451 (51.6)
Working	262 (31.1)	1.309 (51.7)	2.667 (50.3)	421 (48.3)
NA		5 (0.2)	6 (0.1)	1 (0.1)
**Place of ANC**				
Health facility	646 (76.7)	1.999 (79.0)	4.097 (77.3)	668 (76.5)
Non-health facility	112 (13.3)	267 (10.6)	683 (12.9)	122 (14.0)
NA	85 (10.1)	264 (10.4)	520 (9.8)	83 (9.5)
**ANC provider**				
Non-health worker	27 (3.2)	74 (2.9)	193 (3.6)	48 (5.5)
Health worker	731 (86.60)	2.195 (86.7)	4.59 (86.6)	742 (85.0)
NA	86 (10.2)	262 (10.4)	517 (9.8)	83 (9.5)
**Media exposure**				
Not exposed	708 (84)	2.171 (85.8)	4.519 (85.3)	682 (78.1)
Exposed	132 (15.7)	349 (13.8)	746 (14.1)	187 (21.4)
NA	3 (0.4)	9 (0.4)	35 (0.7)	4 (0.5)
**Criteria of ANC**				
Unqualified	664 (78.7)	1.928 (76.2)	4.15 (78.3)	720 (82.4)
Qualified	97 (11.5)	355 (14.0)	645 (12.2)	71 (8.2)
NA	82 (9.7)	247 (9.8)	504 (9.5)	82 (9.4)
**Birth status**				
Premature	52 (6.1)	111 (4.4)	189 (3.6)	39 (4.5)
Normal	791 (93.9)	2.419 (95.6)	5.111 (96.4)	834 (95.5)
**Birth weight status**				
Non-LBW	650 (77.1)	2.003 (79.2)	4.087 (77.1)	646 (74.0)
LBW	57 (6.8)	143 (5.6)	331 (6.3)	48 (5.5)
NA	136 (16.1)	385 (15.2)	882 (17)	179 (20.5)

**Notes:** (4T): “too young” (maternal age ≤ 19 years at the time of last delivery), “too old” (maternal age > 35 years at the time of last delivery), “too close” (birth interval between two last births <24 months) and “too many” (total births > 2 children).

**Abbreviations:** NA, not available; ANC, antenatal care; LBW, low birth weight.

Based on the wealth index, most of the women were in a low wealth index category: women who were “too young” (60%), “too many” (44%), and “too close” (47%). Based on employment status, more than half of the women were not working, namely, women who were “too young” (69%) and “too close” (52%). In addition, more than half of the women underwent pregnancy checks at health facilities, namely women with “too old” (79%), while “too young”, “too much” and “too close” were 77% each. The results of the descriptive analysis also showed that four out of five women had relatively more ANC check-ups with health workers in each 4T category. Table 1 also shows that women who perform qualified antenatal care in each 4T category have a percentage of less than 15%.

Based on the birth status of the children, almost all of them were born at term (normal) with a percentage above 90% in each 4T category. Likewise in all 4T categories, more children were born with non-LBW status (above 90%).

### Qualified ANC in women of childbearing age with 4T

Table 2 shows the percentage distribution of ANC quality among women with 4T in each category according to background characteristics. Most women with a 4T have unqualified antenatal care. Just under 20% of women with a 4T perform qualified ANC. Women with “too old” performed qualified ANC (17%) more than women with other 4T *.*

**Table 2.** Sociodemographic characteristics among women with 4T according to the quality of ANC.

Variables	Too young		Too old		Too many		Too close	
	Unqualified ANC	Qualified ANC	Unqualified ANC	Qualified ANC	Unqualified ANC	Qualified ANC	Unqualified ANC	Qualified ANC
	(%)	(%)	(%)	(%)	(%)	(%)	(%)	(%)
**Education level**								
Low	88.3	11.7	89.9	10.1	90.3	9.7	96.0	4.0
Middle	86.7	13.3	81.6	18.4	83.9	16.1	89.8	10.2
High	90.1	9.9	76.8	23.2	84.1	15.9	87.6	12.4
**Region**								
Urban	85.0	15.0	79.3	20.7	82.0	18.0	88.2	11.8
Rural	88.4	11.6	89.9	10.1	90.7	9.3	93.7	6.3
**Wealth index**								
Low	89.5	10.5	92.8	7.2	92.2	7.8	94.8	5.2
Middle	87.7	12.3	84.9	15.1	87.7	12.3	91.2	8.8
High	78.9	21.1	76.8	23.2	79.2	20.8	86.1	13.9
**Employment status**								
Not working/housewife	86.5	13.5	85.7	14.3	86.3	13.7	91.3	8.7
Working	88.9	11.1	83.2	16.8	86.8	13.2	90.6	9.4
**Place of ANC**								
Health facility	85.6	14.4	83.1	16.9	85.3	14.7	89.6	10.4
Non-health facility	96.1	3.9	93.3	6.7	93.6	6.4	98.5	1.5
**ANC provider**								
Non-health worker	100.0	-	100.0	-	100.0	-	100.0	-
Health worker	86.7	13.3	83.8	16.2	85.9	14.1	90.4	9.6
**Media exposure**								
Not exposed	87.0	13.0	85.4	14.6	86.8	13.2	91.3	8.7
Exposed	88.3	11.7	79.4	20.6	85.1	14.9	89.6	10.4
**Birth status**								
Premature	94.9	5.1	85.5	14.5	91.7	8.3	97.2	2.8
Normal	86.7	13.3	84.4	15.6	86.3	13.7	90.7	9.3
**Birth weight status**								
Non-LBW	86.4	13.6	83.4	16.6	85.2	14.8	90.1	9.9
LBW	86.8	13.2	85.7	14.3	90.9	9.1	92.1	7.9
**Total**	**87.2**	**12.8**	**84.4**	**15.6**	**86.5**	**13.5**	**91.0**	**9.0**

**Notes:** (4T): “too young” (maternal age ≤ 19 years at the time of last delivery), “too old” (maternal age > 35 years at the time of last delivery), “too close” (birth interval between two last births <24 months) and “too many” (total births > 2 children).

**Abbreviations:** ANC, antenatal care; LBW, low birth weight.

Among women with 4T categories, 82% have unqualified ANC. While the higher the education of women, the more women who perform qualified ANC in each 4T category. Most of the women with qualified ANC were found in high education in the “too old” (23%) and “too close” (12%) categories. Meanwhile, in the “too many” and “too young” categories, most of the women with qualified ANC were found in secondary education, 16%, and 13% respectively.

Based on the place of residence, more women who live in urban areas perform qualified ANC for each of the “4T” categories compared to women who live in rural areas. Furthermore, based on wealth status, the higher the wealth index, the more women who perform qualified ANC in each 4T category. Based on employment status, it is seen that women who are not employed are more likely to do qualified ANC at “too young” and “too many” respectively (14%). Meanwhile, among women with “too old” (17%) and “too close” (9%), most of the women with qualified ANC were working. Less than a fifth of “4 Too” women perform qualified ANC at health facilities in each 4T category and all of them are handled by health professionals.

Women who performed qualified ANC were relatively higher among those exposed to information through the media, among women “too old” (21%), “too many” (15%), and “too close” (10%). Meanwhile, when viewed from birth status, women who gave birth to children at term/normally had relatively more qualified ANC for each 4T category compared to women with premature births of their last child. Based on the LBW category, relatively more women with non-LBW babies perform qualified ANC in each 4T category compared to women with LBW babies.

### The incidence of LBW according to the ANC and characteristics of childbearing-age women with 4T

Table 3 shows the results of the logistic regression model testing between the characteristics and quality of ANC variables on the incidence of babies born with LBW in women with 4T. The effect of several variables on the incidence of LBW in each 4T risk model shows mixed results. The quality of the ANC only affects women with “too many” children on bivariate testing or together with other variables. Preterm birth status has a significant influence on the incidence of LBW in all groups of women with 4T compared to the quality of ANC and other variables. Babies born prematurely in the “too close” group of women have the greatest chance of LBW incidence compared to babies born normally in the other 4T category, as well as exposure to information through the media.

**Table 3.** Relationship of ANC in women of childbearing age with 4T on the incidence of LBW.

Variables	Too young		Too old		Too many		Too close	
	AOR (LL-UL)	SOR (LL-UL)	AOR (LL-UL)	SOR (LL-UL)	AOR (LL-UL)	SOR (LL-UL)	AOR (LL-UL)	SOR (LL-UL)
**Education level**								
High	Ref							
Low	0.51 [0.9–2.97]	0.35 [0.76–1.57]	***2.76 [1.30–5.88]	***2.32 [1.26–4.30]	***2.85 [1.65–4.93]	***2.86 [1.77–4.63]	**5.25 [1.27–21.74]	***6.63 [1.88–23.43]
Middle	0.35 [0.06–1.92]	*0.25 [0.56–1.07]	1.22 [0.59–2.51]	1.18 [0.63–2.24]	**1.77 [1.04–3.01]	**1.66 [1.02–2.70]	**3.64 [0.98–13.54]	**4.20 [1.25–14.10]
**Region**								
Rural	Ref							
Urban	1.05 [0.56–1.98]	1.10 [0.63–1.93]	1.39 [0.93–2.08]	1.11 [0.79–1.56]	**1.37 [1.06–1.79]	1.09 [0.87–1.37]	1.25 [0.61–2.55]	0.79 [0.44–1.42]
**Wealth index**								
High	Ref							
Low	1.21 [0.56–2.64]	1.03 [0.53–2.00]	*1.61 [0.97–2.65]	**1.57 [1.07–2.30]	**1.43 [1.05-1.95]	***1.53 [1.19-1.96]	1.25 [0.53-2.92]	1.5 [0.78-2.90]
Middle	**0.19 [0.05–0.71]	***0.18 [0.52–0.64]	1.25 [0.73–2.13]	1.33 [0.83–2.14]	0.83 [0.57-1.20]	0.99 [0.70-1.38]	0.76 [0.28-2.09]	1.25 [0.52-2.96]
**Employment status**								
Working	Ref							
Not working/housewife	0.91 [0.481.71]	0.92 [0.51-1.66]	1.05 [0.72-1.52]	1.04 [0.74-1.46]	0.96 [0.75-1.22]	0.97 [0.77-1.21]	0.75 [0.39-1.45]	0.99 [0.55-1.80]
**Place of ANC**								
Non-health facility	Ref							
Health facility	0.73 [0.31-1.72]	0.77 [0.34-1.73]	1.43 [0.70-2.91]	1.14 [0.60-2.19]	1.08 [0.72-1.64]	0.97 [0.66-1.41]	0.77 [0.26-2.25]	0.59 [0.24-1.48]
**ANC provider**								
Health worker	Ref							
Non-health worker	2.05 [0.42-9.99]	2.29 [0.62-8.50]	2.34 [0.62-8.82]	1.60 [0.52-4.95]	1.56 [0.73-3.35]	*1.73 [0.92-3.27]	1.26 [0.21-7.61]	1.35 [0.30-6.04]
**Media exposure**								
Exposed	Ref							
Not exposed	*2.72 [0.87-8.48]	*2.48 [0.87-7.06]	**0.56 [0.32-0.96]	0.82 [0.51-1.31]	1.04 [0.70-1.55]	*1.36 [0.94-1.96]	**3.96 [1.05-15.00]	**4.40 [1.27-15.08]
**Criteria of ANC**								
Qualified	Ref							
Unqualified	0.78 [0.33-1.82]	1.03 [0.47-2.30]	0.94 [0.56-1.57]	1.19 [0.74-1.93]	*1.47 [0.98-2.20]	***1.74 [1.17-2.56]	0.74 [0.24-2.36]	1.2 [0.43-3.77]
**Birth status**								
Normal	Ref							
Premature	***10.48 [4.74-23.16]	***7.81 [3.84-15.87]	***16.63 [10.42-26.53]	***13.67 [8.79-21.25]	***15.00 [10.76-21.00]	***14.23 [10.31-19.66]	***21.72 [9.23-51.15]	***16.77 [7.85-35.81]

* Significant 0.1.

** Significant 0.05.

*** Significant 0.01.

**Notes:** (4T): “too young” (maternal age ≤ 19 years at the time of last delivery), “too old” (maternal age > 35 years at the time of last delivery), “too close” (birth interval between two last births <24 months) and “too many” (total births > 2 children).

**Abbreviations:** ANC, antenatal care; LBW, low birth weight; SOR, simple odds ratio; AOR, adjusted odds ratio; LL, lower level; UL, upper level.

In women with “too young” status, last childbirth status, women’s exposure to media, and wealth index showed a significant effect when tested per variable or simultaneously on the incidence of LBW babies born. Women with the premature birth of their last child had a 10.48 times greater tendency to give birth to LBW babies compared to women who gave birth to a normal last child (AOR: 10.48; 99% CI; 4.74-23.16). In addition, women who were not exposed to the media had a 2.72 times greater tendency to give birth to LBW babies compared to women who were exposed to the media (AOR: 2.72; 90% CI; 0.87-8.48). Based on social characteristics, women with a middle wealth index were 0.19 times less likely to give birth to LBW babies than women with a high wealth index (AOR: 0.19; 95% CI; 0.05-0.71).

In the second model, “too old”, the status of the last child’s birth, education level, wealth index, and media exposure had a significant effect on the incidence of LBW both on the test per variable and simultaneously. The qualified ANC in the “too old” group of women did not show a significant effect on the incidence of LBW, as well as the area of residence, place of ANC examination, ANC examiner staff, and employment status. As with the previous model, women in the “too old” group with the premature birth of their last baby had a 16.63 times greater chance of giving birth to LBW babies compared to women who gave birth to a normal last child (AOR:16.63; 99% CI; 10.42-26.53). In addition, women with low levels of education have a 2.76 times greater chance of giving birth to LBW babies than women with higher education levels (AOR: 2.76; 99% CI; 1.30-5.88). Women with a low wealth index were more likely (1.60 times) to give birth to LBW babies compared to women with a high wealth index (AOR: 1.61; 90% CI; 0.97-2.65). Not only education level and wealth index, but media exposure in this group also has a significant effect on the incidence of LBW. Interestingly, women who were not exposed to the media were 0.56 times less likely to give birth to LBW babies compared to women who were exposed to the media (AOR: 0.56; 95% CI; 0.87-8.48). In fact, the opportunity is even greater when tested simultaneously with other variables.

In the third model, women with “too many”, birth status, birth rate, ANC quality, wealth index, and area of residence had a significant influence on both the tests per variable and simultaneously. Women with preterm birth had a 15.03 times greater chance of giving birth to a LBW baby compared to women who gave birth normally (not-preterm birth) (AOR: 15.03; 99% CI; 10.76-21.00). Furthermore, women with unqualified ANC were 1.47 times more likely to give birth to LBW compared to women with qualified ANC (AOR: 1.47; 90% CI; 0.98-2.20). Interestingly, women living in urban areas were 1.37 times more likely to have LBW babies than women living in rural areas (AOR:1.37; 95% CI; 1.06-1.79). Furthermore, women with a low wealth index have a 1.43 times greater chance of giving birth to LBW than women with a high wealth index (AOR: 1.43; 95% CI; 1.05-1.95).

Education level becomes an important variable in the group of women with “too many” and “too close”. The higher the education level of women, the lower the tendency to give birth to LBW babies. Women with low and middle education levels in the group of women with “too many” were more likely to give birth to LBW babies by 2.85 times (AOR: 2.85; 99% CI; 1.65-4.93) and 1.77 times (AOR: 1.77; 95 % CI; 1.04-3.01) compared with women with higher education levels.

It was quite different because the quality of the ANC in the fourth model with “too close” did not show a significant effect on the incidence of LBW. In addition to education level, media exposure and preterm birth status were variables that consistently affect the incidence of LBW. Interestingly, preterm birth status has a nearly double chance of developing LBW in this risk group compared to other risk groups. Women with “too close,” where the distance between the last two children was less than two years and gave birth prematurely, had a 21.72 times greater chance of giving birth to LBW babies than normal births in the simultaneous test. Likewise, women who were not exposed to media in the “too close” group had a greater chance of giving birth to LBW babies than other risk groups.

## Discussion

Indonesia has tried to reduce infant mortality. One of the strategies is to prevent the incidence of babies with LBW. The results of this study showed that the birth incidence of LBW babies was almost the same in each 4T category, which is around 6 to 7%. This figure is lower than other Asian countries, such as India. ^18^ While comparing it to African countries, the LBW in Indonesia is lower. ^17^

The results indicate that ANC quality only affects LBW births in the category of too many children. Even so, previous studies also showed a significant relationship between ANC utilization and mothers who were too old (>35 years), whereas mothers who were too old were higher in using ANC. ^20^ However, mothers who were too young had higher knowledge than mothers who were too old. ^21^ A previous study showed that most adolescent births were from mothers with a low education level. ^22^

Women with too many children and unqualified ANC will have a 1.47 times higher chance of giving birth to LBW babies than women with too many children and qualified ANC. This was in accordance with research conducted in Padang, mothers with less than four ANC visits were more likely to give birth to LBW babies compared to mothers with four ANC visits. ^23^ Similarly, studies conducted in India ^18^ and China ^8^ also stated that a comprehensive antenatal examination was associated with a reduced risk of LBW in infants. Studies conducted in Rwanda, ^24^ Ethiopia, ^12^^,^^16^^,^^17^^,^^25^ and Sri Lanka ^11^ found that lack of ANC visits was associated with low infant weight. In a comprehensive ANC, including a complete number of visits, pregnant women carry out regular checkups, practice healthy living habits and obtain iron intake during pregnancy. Thus, they can detect problems, diseases, or complications during pregnancy early, including reducing the incidence of babies with LBW. ^12^^,^^13^^,^^16^^–^^18^^,^^24^^,^^25^

The results of this study showed that the age of childbirth has a significant effect on infants with LBW. Premature birth had the most significant impact on infants with low birth weight in the four 4T categories, namely “too young”, “too old”, “too many”, and “too close”. The World Health Organization (WHO) stated that premature birth is the cause of about one-third of LBW babies. Studies conducted in Yemen ^15^ and Ethiopia ^26^ showed the same. Likewise, in Abu Dhabi, babies born prematurely have an 18 times higher risk of becoming LBW. ^7^ This happens probably because fetal growth and weight gain mainly occur in the late period of pregnancy, so premature babies receive less nutrition which causes low birth weight.

Several socioeconomic and demographic characteristics such as education level, wealth index, and area of residence were significantly associated with the incidence of infants with LBW. The education variable has a significant relationship with the incidence of LBW in all four categories, except for women with births too young, which does not have a relationship with LBW incidence. In line with research conducted by Nuryani and Rahmawati, 2017 in Gorontalo Regency, there was a significant relationship between education level and the incidence of LBW (p=0.017). ^27^ This finding is in agreement with several studies conducted in other developing countries such as India, ^18^ Ethiopia, ^25^ and Ghana. ^28^ Generally, women with higher education were more informed about the risks of not receiving health care during pregnancy and paid more attention to nutritional intake during pregnancy, ^18^^,^^28^^,^^29^ On the other hand, women with low education generally had less access to health facilities, especially economically. ^25^ However, the research conducted by Sharma *et al.* (2015) in Nepal and by Rahim FK and Muharry A (2018) in Kuningan showed different things, where maternal education was not associated with the incidence of LBW. ^30^^,^^31^

Regarding the wealth index in this study, among women with too old and too many, it is seen that low wealth index are more at risk for giving birth to babies with LBW compared to high wealth index. Studies in India ^18^ and Sri Lanka ^11^ showed similar results, where the incidence of LBW decreases with an increasing wealth index.

The residence variable is seen only in the category of too many children, which has a significant effect on LBW births. Women with too many children who live in urban areas are 1.37 times more likely to give birth to LBW babies than women in rural areas. In line with research conducted by Mohammed S *et al.* (2019), the probability of giving birth to an LBW baby was significantly higher in urban residents. ^32^ This is different from the results of the study by Kaur *et al.* (2019) that found that LBW was more common in rural areas than in urban areas (9.8% vs. 2.0%, p=0.03) ^33^ and several studies conducted in Ethiopia. ^16^^,^^17^ This may be related to the education level of women who generally have low and middle education in this study.

## Conclusion

Based on bivariate testing or together with other variables, qualified ANC only has a significant effect on the incidence of LBW in women with the “too many” criteria. It is known that the most influential variable on LBW in women with 4T is premature birth. Besides that, it is known that with a low level of education women who give birth too close have the highest chance of giving birth to LBW compared to the other “4 too” criteria. Likewise, women who are “too old” and “too many” with a low wealth index and women who are “too many” who live in urban areas have the highest chances of giving birth to LBW.

The findings show that the recommendations for qualified ANC according to WHO standards have not been fully implemented. In the case of ANC visits of at least eight times, it is hoped that ANC with health workers at health facilities can be increased. It is also necessary to increase the monitoring of pregnant women with the risk of 4T to continue making ANC visits to reduce the risk of preterm and reduce LBW births. Moreover, increasing education and counseling related to maturing the age of marriage, reproductive health, family planning (spacing), and the dangers of 4T to reduce the risk of LBW events in women with 4T in various information media. It is necessary to review the coverage of ANC in the National Health Insurance (NHI) mechanism, which is only four times, especially for the poor and with low education.

## Data Availability

The dataset of the Indonesia Demographic and Health Survey 2017 is available from the DHS Program website ( https://dhsprogram.com/data/available-datasets.cfm). The data can be obtained after registering and requesting permission to download the dataset through the website. The authors did not have any special access privileges that others would not have.
