# Supplementary material for: Antenatal care utilization on low birth weight children among women with high-risk births
Source: F1000Res. 2024 Jan 24;12:399. Originally published 2023 Apr 13. [Version 2] doi: 10.12688/f1000research.126814.2 (PMC10905146; doi:10.12688/f1000research.126814.2)
Supplement: Peer review history

# Reviewer report: Reviewer response for version 2

**DOI:** 10.5256/f1000research.161763.r240437

**Published:** 2024-02-20

**Authors:** Gouranga Dasvarma

**Affiliations:** 1 College of Humanities, Arts and Social Sciences, Flinders University, Adelaide, SA, Australia

I have gone through the revisions. The revised manuscript may now be accepted for indexing.

Is the work clearly and accurately presented and does it cite the current literature?

Partly

If applicable, is the statistical analysis and its interpretation appropriate?

Partly

Are all the source data underlying the results available to ensure full reproducibility?

Yes

Is the study design appropriate and is the work technically sound?

Partly

Are the conclusions drawn adequately supported by the results?

Partly

Are sufficient details of methods and analysis provided to allow replication by others?

Partly

Reviewer Expertise:

NA

I confirm that I have read this submission and believe that I have an appropriate level of expertise to confirm that it is of an acceptable scientific standard.

# Reviewer report: Reviewer response for version 2

**DOI:** 10.5256/f1000research.161763.r240438

**Published:** 2024-02-16

**Authors:** Garedew Tadege Engdaw

**Affiliations:** 1 Department of Environmental and Occupational Health and Safety, Institute of Public Health, College of Medicine and Health Sciences, University of Gondar, Gondar, Ethiopia

I have no further comments to make for this version.

Is the work clearly and accurately presented and does it cite the current literature?

Partly

If applicable, is the statistical analysis and its interpretation appropriate?

Yes

Are all the source data underlying the results available to ensure full reproducibility?

Yes

Is the study design appropriate and is the work technically sound?

Yes

Are the conclusions drawn adequately supported by the results?

Partly

Are sufficient details of methods and analysis provided to allow replication by others?

Yes

Reviewer Expertise:

Public health and Epidemiology

I confirm that I have read this submission and believe that I have an appropriate level of expertise to confirm that it is of an acceptable scientific standard.

# Reviewer report: Reviewer response for version 1

**DOI:** 10.5256/f1000research.139258.r189795

**Published:** 2023-08-17

**Authors:** Garedew Tadege Engdaw

**Affiliations:** 1 Department of Environmental and Occupational Health and Safety, Institute of Public Health, College of Medicine and Health Sciences, University of Gondar, Gondar, Ethiopia

The title is very useful and interesting. The paper needs major revision - below are my recommendations and questions for the development of the paper:

1. The sentences require editing in grammar. Needs checking grammar and should be re-edited.
2.  WHO recommends 4 or more ANC visits including 1st ANC in the first trimester. However, the authors focused on the effect of ANC visit on LBW and sociodemographic factors with women on high-risk birth, regardless the number of ANC visits. Some women visit a health facility for ANC just before the delivery. If the authors could compare the effect of LBW among women with no ANC visit to the women with 4 or more ANC visits, the results might be more plausible (NA).
3. For the analysis, you provided the definition of LBW to include the literature. You also need to provide the definition of ANC visits for inclusion criteria. If one or more ANC visits were your inclusion criteria, your conclusion may mislead the readers. You need to explain why you selected literatures mentioning ANC visit only, not the number of ANC visits in the limitation section.
4. **Education level**: It is the levels such as preschool, primary school, lower secondary school, upper secondary and higher education (diploma, certificate and above). Better to write the educational level of women’s in a scientific way, unless you have evidence for such classification from table 1. Make words uniform across the document (like Educational level from table …Low, Middle High, while from the prose it says secondary. It lacks consistency).
5. Your outcome of interest is to see the effect of ANC and socio demographic factors on LBW, however from Table 1 you presented the birth weight status of women’s as NA (what is the importance of presenting this result, if the status is already unknown?).
6. Operationalize the word **Qualified ANC** and **Non-Qualified ANC,** **Non-health worker in ANC provider **women of childbearing age.

**Discussion:**

1. From the first paragraph you wrote as “This figure is lower than other Asian **countries**, such as India. ^18^ While comparing it to African **countries**, the LBW in Indonesia is lower”. Make a correction for this paragraph as in “Asian country” and “African country” - you had only one literature for this evidence. Works for the whole document.

You think the two sentences had difference? This figure is **lower than** other Asian countries, such as India. ^18^ While comparing it to African countries, the LBW in Indonesia is **lowe** **r**” (the figure is “ **lower than both in Asian country** **and Indonesia”…) **

1. Write the possible reason for the discrepancy (in your study and other studies) including the factors for the discussion.
2. From the last paragraph of the discussion you wrote as “ **several** studies conducted in Ethiopia. ^16, 17^ This may be related to the education level of women who generally have low and middle education in this study". You had only two evidences. Generally speaking, 'several' is used to refer to quantities above two or so but not so much that it's a lot or many. Perhaps the most common interpretation or intended sense of several is around three to five, but this can vary greatly depending on the context. Change it to 'some studies'.

**Conclusion: **

1. In the case of ANC visits of at least **eight times**, *it is hoped that ANC with health workers at health facilities can be increased*. Better to conclude your findings based on your discussion (the number of ANC visit is not related with your study/objective).
2. It is necessary to review the coverage of ANC in the National Health Insurance (NHI) mechanism, which is only four times, especially for the poor and with low education. Not related with your objective.

How is reviewing the National Health Insurance (NHI) related with LBW and related with ANC? It may be showing only the ANC attendants, number of ANC, the service provided during each ANC…not the low birth weight risks of the mothers on ANC follow up **(remove the above two sentences).**

Is the work clearly and accurately presented and does it cite the current literature?

Partly

If applicable, is the statistical analysis and its interpretation appropriate?

Yes

Are all the source data underlying the results available to ensure full reproducibility?

Yes

Is the study design appropriate and is the work technically sound?

Yes

Are the conclusions drawn adequately supported by the results?

Partly

Are sufficient details of methods and analysis provided to allow replication by others?

Yes

Reviewer Expertise:

Public health and Epidemiology

I confirm that I have read this submission and believe that I have an appropriate level of expertise to confirm that it is of an acceptable scientific standard, however I have significant reservations, as outlined above.

# Reviewer report: Reviewer response for version 1

**DOI:** 10.5256/f1000research.139258.r170911

**Published:** 2023-05-25

**Authors:** Gouranga Dasvarma

**Affiliations:** 1 College of Humanities, Arts and Social Sciences, Flinders University, Adelaide, SA, Australia

General comments:

1. This is a useful study of the determinants of low birth weight babies in Indonesia based on data collected at a recent national survey, namely The Indonesia Demographic Health Survey 2017.
2. The authors have approached the problem of low birth weight babies (LBW) by sensibly selecting the groups of women who are at the highest risk of giving birth to babies with less than the recommended weight of 2,500 grams. Such groups of women comprise those with the following characteristics at the birth of their children, namely women (i) who are too young (less than 20 years of age), (ii) too old (more than 35 years of age), (iii) have too many children (3 or more) and too close (birth interval less than 2 years).
3. The aim of the study is to examine the effects of antenatal care (ANC) on the prevalence of LBW in each of the high-risk groups of women mentioned above, with the hypothesis that qualified ANC reduces the risk of LBW babies in the high-risk women.
4. The justification of the study appears to be that, by assumption the prevalence of LBW is high in the high-risk groups of women and that it can be reduced by good ANC. However, the prevalence of LBW is 7.1% as at the 2017 Indonesia Demographic and Health Survey, and it seems that Indonesia may be on track to achieving a 30% reduction in LBW between 2015 and 2030 as one of the Sustainable Development Goals of the United Nations. Therefore, further justification is needed for the present study.
5. Moreover, a similar study, based on data from the 2017 Indonesia Demographic and Health Survey exists (see Safitri *et al.*, 2022 ^1^), which identifies ANC as a determinant of LBW in Indonesia, although the present manuscript focuses on low birthweight among high-risk groups of women. But reference should be made to the Safitri *et al.* study.
6. The manuscript needs a major revision, particularly a revision of Table 2 and rewriting the discussion of Table 2 findings.
7. The manuscript also requires a thorough editing for English.
8. Several other, specific comments are made in the body of the text, which is returned for revision - please also find two attachments of the manuscript with my comments linked here ( Attachment 1 and Attachment 2).

Specific comments:

1. Title. A similar study based on data from the 2017 Indonesia Demographic and Health Survey exists (see Safitri *et al.*, 2022 ^1^), which identifies ANC as a determinant of LBW in Indonesia. Acknowledgement and appropriate references should be made of the Safitri *et al.* study.

What new information does your study provide to the field of knowledge? Is it the analysis of low birthweight among high-risk groups of women?

1. Abstract. Background, Line 2. Rewrite as "and is a leading cause of neonatal mortality".
2. Abstract. Methods, Line 3. "unit of analysis".
3. Abstract. Methods. Line 3. pre-term birth is not included in the four criteria (4Ts) mentioned above.
4. Abstract. Conclusions, Lines 1-2. The sentence reads as if WHO has found that qualified ANC standards have not been fully implemented (in Indonesia). But it is a finding of your analysis, is it not?
5. Methods. Lines 11-12. How was quality of ANC (qualified ANC) determined? There are no data in the 2017 IDHS about the quality of ANC.
6. Methods. Line 12. Do you mean "positive pregnancy experience"?
7. Methods. Line 12. Do you mean to say, "Data were collected at IDHS 2017 only for five WHO recommendations"?
8. Methods. Line 14. All four Ts together?
9. Results. Line 3. The "too many" and "too old" categories of women (as well as the "too close" category") also have high proportions in the High education category. This is notable.
10. Results. Lines 3-4. This is not correct. Please rewrite this part as ""the majority of women in the "too young" and "too many" categories are rural dwellers, almost equal proportions of women in the "too close" category are urban and rural dwellers, and the majority of women in the "too old" category are urban dwellers."
11. Results. Line 6. This is true only for the "too young" women. Please re-write correctly.
12. Results. Lines 10-11. Where is it shown that “four out of five women had relatively more ANC check-ups”?
13. Results. Lines 11-12. How is this true? The table shows that 85% or more of the women each category had a Health worker as their ANC provider. Do you mean to say that most of the Health workers are not qualified?
14. Results. Lines 13-14. This result contradicts your hypothesis that women in the 4T categories run the risk of giving birth to babies with pre-maturity and low birth weight.
15. Table 1. Usually, the dependent variable (in this case women in each criterion group) should be shown on the horizontal axis and the independent variables on the vertical axis. This can be done by formatting the layout of the table as landscape.  Also, try to put the entire table on one page (i.e. do not split a table between pages. Reduce the font size if needed).
16. Table 2. Line 1. "ANC quality" or qualified and unqualified ANC? The two terms are different.
17. Results. First paragraph after Table 1. **Table 2, as presented here shows the distribution of each socio-economic characteristic (independent variable) according to qualified ANC and unqualified ANC for each of the 4T categories of women. But in actual fact, you should show the distribution of qualified ANC and unqualified ANC according to each socio-economic characteristic.  In other words,  show the column percentages instead of row percentages. Therefore, please re-do Table 2 and re-write its description. Please also show the association (chi-square) between each of the socio-economic characteristics and unqualified and qualified ANC for each of the 4T categories.**
18. Table 3. It appears that you have not used any information derived from Table 2 in performing your analysis in Table 3. Please re-calculate Table 2 as suggested and use the relevant information from that (revised Table 2) to select the pertinent variables for logistic regression (Table 3).
19. Results. Table 3, Lines 2-3. The "mixed" results may be due to the effects of confounding factors. For example, take any one category, such as "Too old" - While this group excludes women who are "Too young", the "Too old" women may have children who are too closely spaced or may have too many children. Similarly, the women who have "Too many" children may be "too old" themselves or may have children that are "too closely" spaced, or the women who are in the "Too close" category may have too many children or may be too old themselves. Only the "Too young" women would not be subjected to confounding factors like too many or too old, but they may still have too closely spaced children. It is for these reasons that you should also analyse the group of women who are Too old AND Too Close AND Too Many. The "Too young" group may be analysed separately, because "too young" women would have very little chances of having too many children or too closely spaced children.
20. Results. Table 3, Line 3. "Quality of ANC". Which variable(s) indicate the quality of ANC?
21. Results. Table 3, Line 15 and Line 28. Why do you refer to the groups as "Model"? Just call them what they are i.e., "Too old" or "Too many". A model may have the connotation of a separate logistic regression.
22. Discussion. Line 1. LBW. Is LBW a major cause of infant death in Indonesia?
23. Discussion. Line 1. Indonesia has already reduced its IMR by much, but it is pursuing further declines in IMR.
24. Discussion. Lines 4-5. If the prevalence of LBW is already so low in Indonesia (6-7%), then why study it? You should cite the target of LBW in Indonesia or cite the prevalence of LBW in countries which is lower than that in Indonesia and then justify your study.
25. Discussion Line 5. So, the results show that your hypothesis (that ANC quality affects the prevalence of LBW) is not true in three out of four categories of women. How do you explain this?
26. Discussion. Line 8. What kind of knowledge?
27. Conclusion. Lines 1-2. Assuming that the headings of your Table 1 are correct, according to the numbers of women in each category, women with too many children number 5,300. Thus, qualified ANC affects 55.5% of the women at risk (the number of women with all the Ts is equal to 9,546).
28. Conclusion. Line 8. Re ANC visit of at least eight times. The recommendation of eight ANC visits from the WHO came out in 2016 and probably implemented in Indonesia after the 2017 IDHS was conducted. Therefore, in most cases, at the time of IDHS 2017 the recommendation was to have at least 4 ANC visits. Table 9.2 of the 2017 IDHS Final report shows 90.6% of the women giving birth in the last five years had 4+ ANC visits.

Recommendation:

 The authors should address the comments, provide further justification of this study and submit a revised manuscript.

Is the work clearly and accurately presented and does it cite the current literature?

Partly

If applicable, is the statistical analysis and its interpretation appropriate?

Partly

Are all the source data underlying the results available to ensure full reproducibility?

Yes

Is the study design appropriate and is the work technically sound?

Partly

Are the conclusions drawn adequately supported by the results?

Partly

Are sufficient details of methods and analysis provided to allow replication by others?

Partly

Reviewer Expertise:

Demography, including infant and child mortality, maternal mortality, fertility, population and development, population and environment

I confirm that I have read this submission and believe that I have an appropriate level of expertise to confirm that it is of an acceptable scientific standard, however I have significant reservations, as outlined above.

## References

[1] : Determinant factors of low birth weight in Indonesia: Findings from the 2017 Indonesian demographic and health survey. *J Pediatr Nurs* .2022;63: 10.1016/j.pedn.2021.10.005 e102-e106 10.1016/j.pedn.2021.10.005 34688529
